# Angiopoietin‐1, Angiopoietin‐2 and Vascular Endothelial Growth Factor Levels in Canine Leishmaniosis

**DOI:** 10.1002/vms3.70871

**Published:** 2026-03-14

**Authors:** Gamze Gultekin, Erdogan Malatyali, Evren Tileklioglu, Hatice Ertabaklar, Songul Erdogan, Gamze Sevri Ekren Asici, Asude Gulce Oryasin, Serdar Pasa, Kerem Ural, Hasan Erdogan, Mehmet Gultekin

**Affiliations:** ^1^ Central Laboratory Faculty of Veterinary Medicine Aydin Adnan Menderes University Aydin Turkey; ^2^ Department of Parasitology Faculty of Medicine Aydin Adnan Menderes University Aydin Turkey; ^3^ Department of Internal Medicine Faculty of Veterinary Medicine Aydin Adnan Menderes University Aydin Turkey; ^4^ Department of Biochemistry Faculty of Veterinary Medicine Aydin Adnan Menderes University Aydin Turkey; ^5^ Department of Parasitology Faculty of Veterinary Medicine Aydin Adnan Menderes University Aydin Turkey

**Keywords:** angiopoietins, biomarkers, dogs, inflammation, leishmaniosis

## Abstract

**Background:**

Canine leishmaniosis (CanL) is a zoonotic vector–borne disease and is primarily associated with systemic inflammation, endothelial dysfunction and immune dysregulation. Although angiopoietins (Ang) and vascular endothelial growth factor (VEGF) are key regulators of vascular homeostasis, their roles in CanL have remained unclear.

**Objectives:**

The present study aimed to evaluate Ang‐1, Ang‐2 and VEGF levels in CanL, in relation to disease severity and inflammatory markers.

**Methods:**

The study included 74 dogs (52 with CanL and 22 healthy controls). The dogs with CanL were categorized into four groups based on disease severity. Plasma levels of Ang‐1, Ang‐2 and VEGF were measured with commercial ELISA kits. The correlations of these parameters with disease progression and inflammatory markers, such as C‐reactive protein (CRP) and ferritin, were statistically analysed.

**Results:**

Ang‐1 and VEGF levels were significantly elevated in advanced stages of CanL, suggesting a compensatory response to vascular damage and chronic inflammation. Ang‐2 levels increased in both early and late stages, indicating endothelial destabilization and inflammatory progression. VEGF correlated with disease severity and aligned with enhanced vascular permeability. CRP and ferritin levels increased with disease severity and reflected systemic inflammation.

**Conclusions:**

This study highlights the potential role of VEGF, Ang‐1 and Ang‐2 in CanL progression. High levels of VEGF and Ang‐2 suggest enhanced vascular permeability and inflammation, whereas elevated Ang‐1 may indicate a compensatory response. Overall, these parameters have the potential to be used as biomarkers for monitoring disease progression and as targets for therapy. Additional studies with larger cohorts and advanced imaging techniques are needed to validate these findings.

## Graphical abstract

Ang‐1, Ang‐2 and VEGF levels significantly varied in different stages of canine leishmaniosis.

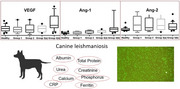

## 1 Introduction

The genus Leishmania comprises a diverse group of parasitic species that can infect a wide range of mammalian hosts, including humans. Phlebotomine sandflies, widely distributed nocturnal insects, are the primary vector of the parasite. The parasite is transmitted between hosts during the blood‐feeding activities of sandflies; transplacental and venereal transmission has also been reported in dogs (Boggiatto et al. 2011). Over 70 mammalian species have been identified as natural reservoir hosts of the parasite; however, dogs play the most significant role due to their close contact with humans and shared habitats (Esteva et al. 2017). Leishmaniosis is a growing public health concern in many EU and neighbouring countries, as global warming has expanded the habitat of sandflies. However, comprehensive surveillance and control programmes for both animal and human infections are still not implemented in many countries (Berriatua et al. 2021).

Canine leishmaniosis (CanL) is a multi‐systemic, chronic disease of dogs. The clinical findings can range from asymptomatic carriage to severe, life‐threatening disease, particularly with renal and cardiovascular involvement (Gultekin et al. 2023). Amastigote form of the parasite drives the pathogenesis, affecting tissues and immune‐mediated inflammatory processes, including the production of autoimmune antibodies and immune complex deposition (Solano‐Gallego et al. 2009; Paltrinieri et al. 2010). In addition to parasite‐related factors, vector gut microbiota and yellow proteins in sandfly saliva are related to the promotion of the infection through host interleukin (IL)‐1β amplification and neutrophil recruitment (Serafim et al. 2021). Inflammatory mechanisms can lead to glomerulonephritis, tubulointerstitial nephritis and amyloidosis, along with systemic complications such as vasculitis and myocardial damage (Roura et al. 2021; de Sousa et al. 2025). Overall, these processes significantly increase the severity of CanL, which can result in granulomatous inflammation, proteinuria and hypertension (Koutinas and Koutinas 2014).

Previous studies have highlighted the significance of vascular endothelial dysfunction and angiogenesis in the pathophysiology of CanL (Fraga et al. 2012; Weinkopff et al. 2019; Ribeiro et al. 2024). Angiopoietin‐1 (Ang‐1) and angiopoietin‐2 (Ang‐2) are key regulatory proteins that modulate vascular stability and permeability, whereas vascular endothelial growth factor (VEGF) promotes the proliferation and migration of endothelial cells (Akwii et al. 2019; König et al. 2019; Zhao et al. 2025). In addition, elevated Ang‐2 levels were associated with disease prognosis in myxomatous valve degeneration, splenic hemangiosarcoma and systemic inflammation in dogs (König et al. 2018; Brunner et al. 2021; Wongsuttawas et al. 2021; Yu et al. 2024).

In humans, endothelial angiopoietin pathway proteins, such as Ang‐2 blocking antibodies, are considered key therapeutic targets for various diseases, including cancer, diabetes, ocular disorders, atherosclerosis and complications related to organ transplantation (Saharinen et al. 2017). In addition, both Ang‐1 and Ang‐2 were offered as possible targets for the treatment of malaria as the plasma levels were correlated with the severity of the disease (de Jong et al. 2016). The investigation of plasma Ang‐1, Ang‐2 and VEGF levels in CanL has great potential for diagnostic and prognostic insights and supports the development of novel therapeutic targets. Despite the fact that impairment of epithelial and endothelial barrier function is often associated with infectious diseases, the importance of these parameters is debated in the literature on CanL. Studies examining these parameters together in dogs have remained limited to systemic inflammatory response syndrome (SIRS) and sepsis (König et al. 2018, 2019). This study aims to analyse the diagnostic and prognostic potential of certain endothelial regulator proteins in CanL by examining the plasma levels of Ang‐1, Ang‐2 and VEGF in dogs at different clinical stages of the disease.

## 2 Materials and Methods

### 2.1 Ethical Statement

The study was reviewed and approved by the Local Ethics Committee for Animal Experiments at Aydin Adnan Menderes University (64583101/2020/018). Informed consent was obtained from dog owners before participation in the study.

### 2.2 Study Design and Subjects

A total of 74 dogs, 52 dogs with CanL and 22 healthy dogs, were included in this prospective observational study. Samples and clinical data were collected from July 2021 to January 2023. Dogs with a prior history of treatment for CanL or other chronic diseases, either at our facility or at another institution, were excluded from the study. Dogs of varying ages, medium‐sized breeds and both sexes were grouped according to the clinical and laboratory findings. Dogs with CanL were classified into four groups on the basis of clinical findings, haematological and biochemical parameters, renal function tests, antibody titres, acute phase protein levels, C‐reactive protein (CRP) and ferritin, as previously described by Ceron et al. (2018). The first group (G1) consisted of dogs without clinical signs, with normal acute phase protein levels, and confirmed infection based on serological or molecular tests. The second group (G2) included dogs with mild clinical signs and slight elevations in acute‐phase protein levels. The last groups (G3a and G3b) consisted of dogs with moderate clinical signs and moderate increases in acute phase proteins, whereas G3b included those with severe clinical signs, significant immune complex disorders and marked increases in acute phase protein levels. G1–G3 groups were composed of dogs presented to our clinic either with various clinical complaints or for routine health screenings.

The control group consisted of dogs that visited the clinic for routine vaccination or health screening. The inclusion criteria were as follows: no pathological findings in haematological, biochemical and parasitological examinations and no clinical signs of CanL. In addition, the age, gender and breed distribution of healthy dogs were matched to those diagnosed with CanL.

### 2.3 The Clinical and Laboratory Diagnosis of CanL

The diagnosis of CanL‐suspected dogs relied on both clinical symptoms and laboratory tests: The detection of amastigote forms in Giemsa‐stained smears of lymph node aspirates and/or a positive result with a commercial immunochromatographic rapid test, SNAP *Leishmania* (Idexx Laboratories, Westbrook, USA), were considered confirmatory for leishmaniosis (Athanasiou et al. 2014). The diagnosis of CanL was also confirmed with an in‐house indirect fluorescent antibody test (IFAT) as previously reported (Ertabaklar et al. 2005). First, a previous local isolate of *Leishmania infantum* MON‐1 was cultured in RPMI‐1640 medium supplemented with 10% foetal calf serum (FCS) at 26°C. Promastigotes at the logarithmic phase were washed eight times with phosphate‐buffered saline (PBS). The final concentration was adjusted to 2 × 10^6^ promastigotes/mL in PBS. Finally, 10 µL of parasite suspension was distributed onto Teflon slide wells and air‐dried at room temperature. The parasite‐coated slides were stored at −20°C until needed for analysis. Before the assay, the slides were brought to room temperature. After incubation with serial dilutions of canine sera (1:32, 1:64, 1:128, 1:256 and 1:512) at 37°C for 30 min, the slides were washed with PBS and incubated with anti‐dog IgG (whole molecule)–FITC antibody (Sigma, Darmstadt, Germany) at 37°C for an additional 30 min. Finally, the slides were covered and examined under a fluorescent microscope (BioSystems, Barcelona, Spain). A titre of 1:64 was defined as the cut‐off point for a positive result.

The presence of other common vector–borne infections, including *Ehrlichia* sp., *Anaplasma* sp. and *Dirofilaria immitis*, was tested using a commercial rapid test kit (Idexx Laboratories, Westbrook, USA) and by examining blood smears. Only CanL‐positive dogs were included in the study.

### 2.4 Evaluation of Biochemical Parameters

Blood samples were collected from dogs after a minimum of 8 h of fasting to reduce metabolic variability related to food intake. Tubes (Greiner Bio‐One, Frickenhausen, Germany) without anticoagulants were used to take blood samples from the cephalic vein of each subject. The tubes were centrifuged, and the separated sera were stored at −20°C until analysis. Serological analyses of urea, creatinine, calcium, phosphorus, total protein and albumin were performed using commercial kits (Randox Laboratories, County Antrim, UK). CRP and ferritin levels were quantified to determine inflammation severity using ELISA kits (Bioassay Technology Laboratory, Shanghai, China). Angiopoietin‐1 (Ang‐1), Angiopoietin‐2 (Ang‐2) and VEGF‐A were measured in all serum samples using canine‐specific ELISA kits (MyBiosource, San Diego, USA; R&D, Minneapolis, USA).

### 2.5 Statistical Analysis

The present data were analysed with IBM SPSS Statistics for Windows, version 29.0 (IBM Corp., Armonk, NY, USA). Data distribution was assessed for normality, and non‐parametric tests were applied. The parameters between the groups were compared using the Kruskal–Wallis test. The results were presented as median (IQR25–IQR75), and the statistical significance level was set at *p* < 0.05.

## 3 Results

The demographics and number of dogs categorized based on acute phase protein changes and including a healthy control group are detailed in Table 1. No significant differences were found between the groups in terms of age and gender (*p* > 0.05).

**TABLE 1:** The numbers and demographics of dogs in the groups.

	Male	Female	Mean age ± SD	Total
G1	5	4	5.33 ± 2.83	9
G2	6	3	6.78 ± 3.49	9
G3a	5	7	7.25 ± 2.95	12
G3b	10	12	8.00 ± 3.12	22
Healthy control	11	11	6.95 ± 2.81	22
Total	37	37		74

The most frequent clinical symptoms in CanL group dogs were cachexia, lethargy, weight loss and peripheral lymphadenopathy in G2, G3a and G3b. Dermatological findings included hypotrichosis, alopecia, ulcerative dermatitis and onychogryphosis, with increasing severity across the groups. Prominent ocular manifestations, including keratitis and conjunctivitis, were observed particularly in G3a and G3b.

Parasitological and biochemical parameters showed significant changes across different stages of CanL (Table 2). IFAT titres were significantly higher in G3b (256 [128–512]) compared to other groups (*p* < 0.05). Albumin levels were highest in the healthy group (2.6 mg/dL [2.5–2.8 mg/dL]) and significantly decreased in the patient groups (*p* < 0.05). Total protein levels increased notably in the patient groups, especially in G1 and G3b (*p* < 0.05). Late disease stages showed significant increases in urea (243 mg/dL [73–442 mg/dL]) and creatinine (3.3 mg/dL [1.5–6.9 mg/dL]) in Group 3b (*p* < 0.05). Calcium levels were highest in the healthy group (10.7 mg/dL [10.3–11.2 mg/dL]) and significantly reduced in all patient groups (*p* < 0.05), whereas phosphorus levels were significantly elevated in G3b (6.3 mg/dL [4.3–11.9 mg/dL]) (*p* < 0.05). CRP (26.3 µg/mL [22–58 µg/mL]) and ferritin (254 ng/mL [178–372 ng/mL]) levels increased progressively with disease severity, with a peak in G3b (*p* < 0.05).

**TABLE 2:** Comparison of IFAT titres and biochemical parameters between dogs with CanL and healthy control group, median (IQR25–IQR75).

Parameter	Healthy	G1 (*n* = 9)	G2 (*n* = 9)	G3a (*n* = 12)	G3b (*n* = 22)	*p* value
IFAT	—^a^	64^b^ (64–128)	64^b^ (64–128)	64^b^ (64–128)	256^c^ (128–512)	<0.001
Albumin (mg/dL)	2.6^a^ (2.5–2.8)	2.3^b^ (2.5–2.8)	2.2^b^ (2.5–2.8)	2.4^b^ (2.5–2.8)	2.2^b^ (2.5–2.8)	0.028
Total protein (mg/dL)	5.9^a^ (5.6–6.1)	8.4^b^ (6.3–9.4)	7.7^b^ (6.8–10.3)	7.0^b^ (6.5–9.1)	7.5^b^ (6.3–8.7)	<0.001
Urea (mg/dL)	33^a^ (23–36)	29^a^ (24–40)	38^a^ (30–51)	33^a^ (23–39)	243^b^ (73–442)	<0.001
Creatinine (mg/dL)	0.9^a^ (0.7–1.1)	0.8^a^ (0.7–1.1)	1.1^a^ (0.7–1.2)	0.9^a^ (0.7–1.1)	3.3^b^ (1.5–6.9)	<0.001
Calcium (mg/dL)	10.7^a^ (10.3–11.2)	9.7^b^ (8.9–9.9)	9.6^b^ (8.5–9.9)	9.3^b^ (9.1–9.9)	9.9^b^ (10.7–12.0)	<0.001
Phosphorus (mg/dL)	5.3^a^ (4.6–5.9)	4.1^a^ (3.1–5.0)	4.1^a^ (3.3–4.3)	4.9^a^ (3.8–5.5)	6.3^b^ (4.3–11.9)	<0.001
CRP (µg/mL)	3^a^ (1.8–6.0)	6.8^ab^ (3.9–10)	15.6^b^ (13.9–17.7)	20.8^c^ (14.1–21.5)	26.3^c^ (22–58)	<0.001
Ferritin (ng/mL)	62^a^ (46–74)	66^ab^ (39–86)	129^b^ (76–151)	124^b^ (87–181)	254^c^ (178–372)	<0.001
VEGF (pg/mL)	132^a^ (88–173)	111^a^ (67–159)	106^a^ (61–203)	135^a^ (67–184)	198^b^ (149–378)	0.014
Ang‐1 (ng/mL)	110^a^ (104–128)	127^ab^ (84–204)	248^bc^ (127–265)	252^cd^ (157–288)	361^de^ (222–451)	<0.001
Ang‐2 (ng/mL)	1330^a^ (1197–1669)	2544^b^ (1132–4767)	1871^ab^ (1226–4347)	2438^b^ (1398–4066)	2025^b^ (1472–2908)	0.025

*Note*: Medians within the same row that share at least one superscript letter do not differ significantly, whereas medians with entirely different letters (a–e) are significantly different (*p* < 0.05). G1–G3 refers to the clinical stage of CanL.

Abbreviations: CanL, canine leishmaniosis; CRP, C‐reactive protein; IFAT, indirect fluorescence antibody test; IQR, interquartile range.

**FIGURE 1:**
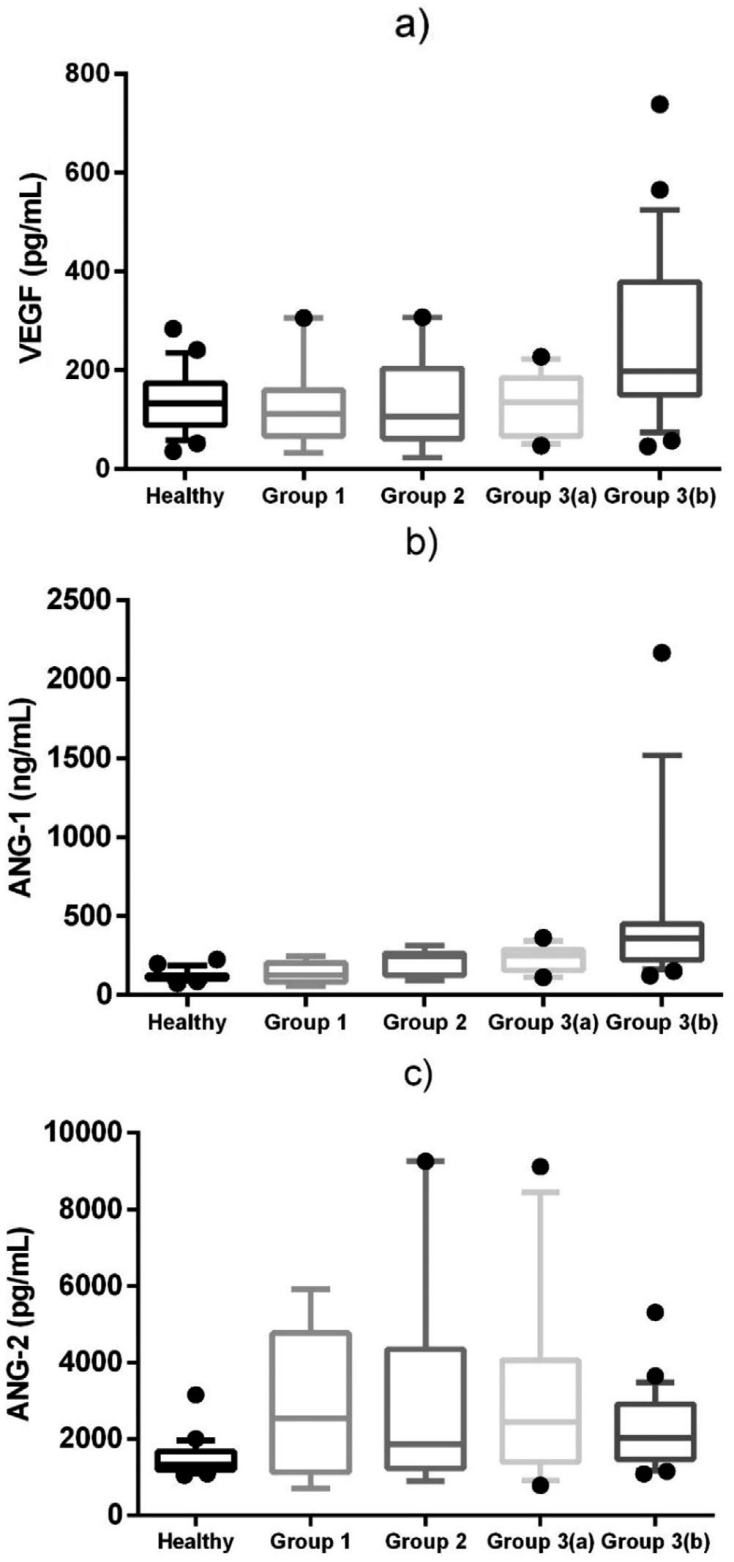
Concentrations of VEGF (a), ANG‐1 (b) and ANG‐2 (c) in dogs with leishmaniosis and healthy dogs. Boxes show the 25th and 75th percentiles, whiskers show the 10th and 90th percentiles. The line inside each box indicates the median. Dots represent results outside the slice. VEGF, vascular endothelial growth factor.

Levels of VEGF, Ang‐1 and Ang‐2, markers of angiogenesis and vascular changes, exhibited significant alterations in the late stages of the disease (Table 2; Figure 1). VEGF levels were notably higher in G3b (198 pg/mL [149–378 pg/mL]) compared to other groups (*p* < 0.05). Ang‐1 levels increased progressively with disease severity, peaking at 361 ng/mL [222–451 ng/mL] in G3b (*p* < 0.05). Ang‐2 levels were significantly elevated in patient groups compared to the healthy group, with marked increases observed in G1 and G3b (*p* < 0.05).

## 4 Discussion

CanL is one of the most important vector–borne diseases, with significant veterinary and public health implications, particularly in Asia, the Americas and the Mediterranean basin. Many biochemical markers related to the disease pathogenesis have been reported in the literature (Maia and Campino 2018). This study was designed to analyse certain angiogenesis and vascular homeostasis protein levels in dogs with CanL. The analysis of these parameters in dogs at different stages of the disease provides a better understanding of the vascular and inflammatory processes in CanL.

The most significant result of this study was that the dogs with CanL showed significantly elevated serum levels of Ang‐1, Ang‐2 and VEGF compared to controls. Ang‐1 levels were particularly elevated in the late stages of the disease (G3a and G3b). Angiopoietin‐1/Tie‐2 receptor activation enhances vascular stability, preserves endothelial barrier integrity and suppresses the inflammatory response (Fiedler and Augustin 2006). This increase likely represents a compensatory mechanism to maintain vascular stability and control inflammation in response to the chronic nature of the disease. Following our findings, Ang‐1 played a protective role against vascular permeability and systemic inflammation in other chronic inflammatory conditions (Costa et al. 2007; Jeong et al. 2021). Angiogenic and renal biomarkers such as Ang‐1, Ang‐2 and VEGF have gained prominence in both veterinary and human research. Chrząszcz et al. (2024) demonstrated decreased Ang‐1 levels in patients with macular neovascularization, pointing to impaired vascular stability. Similarly, Turki and Kasim (2023) found significantly reduced Ang‐1 levels in severe COVID‐19 cases, reinforcing its association with systemic endothelial dysfunction. In contrast, our study identified elevated Ang‐1 levels in advanced CanL, suggesting a compensatory mechanism aimed at restoring vascular integrity during chronic inflammation. From a renal perspective, Luo et al. (2024) and Yildiz et al. (2024) reviewed the prognostic value of Ang‐2 in both acute and chronic kidney disease, emphasizing its predictive capacity for renal outcomes and vascular complications. Complementing these findings, Gultekin and Ulutas (2024) reported elevated FGF‐23 levels in dogs with advanced CanL, correlating with serum urea and creatinine levels, thus supporting the involvement of vascular‐renal interactions in CanL pathophysiology. Taken together, these cross‐species and cross‐system observations highlight the translational and integrative value of angiogenic and renal biomarkers. Targeting the Ang–Tie2–VEGF axis and associated pathways may thus offer promising avenues for monitoring and managing vascular and renal complications in CanL. The elevated Ang‐1 levels in advanced stages suggest a prolonged inflammatory process and a systemic effort to stabilize the vasculature (Eklund and Saharinen 2013). In contrast, no significant changes in Ang‐1 levels were observed in the early stages (G1 and G2) of the disease, suggesting that vascular stability remained intact during these stages, with Ang‐1 release becoming more prominent as inflammation progresses. This pattern was consistent with findings that were reported in other infectious and inflammatory diseases in humans, where Ang‐1 levels correlated with disease severity and vascular integrity (Clavel et al. 2007; Ricciuto et al. 2011). In addition, a 2021 study from Pugliese et al. (2021) found significant increases in SHp levels and negative correlations between ROMs and red blood cell count, as well as between HMGB‐1 and SHp, indicating that oxidative damage contributes to the progression of CanL. The dogs in this previous study showed a significant reduction in parasite load and changes in inflammatory and renal biomarkers following leishmaniosis treatment. The ratio of urinary amylase to creatinine gave promising results for monetarization of treatment response and disease severity at diagnosis (Pantaleo et al. 2024). Circulating immune complexes (CIC) were identified as a promising biomarker candidate for assessing clinical progression, treatment efficacy and relapse detection in CanL (Sarquis et al. 2024).

Ang‐2 levels, unlike VEGF and Ang‐1, significantly increased in both the early and late stages of the disease (G3a and G3b). Ang‐2 disrupts vascular homeostasis by antagonizing the effect on stabilization of the Ang‐1/Tie‐2 receptor complex, increasing vascular permeability and facilitating inflammatory cell infiltration (Akwii et al. 2019). The disruption of the vascular endothelial barrier is a hallmark of uncontrolled inflammation, and elevated Ang‐2 levels have been strongly associated with the exacerbation of vascular dysfunction in chronic inflammatory conditions (Mansour et al. 2022). The increase in the Ang‐2 level may reflect its critical role in the promotion of vascular destabilization and systemic inflammation. Furthermore, Ang‐2 has a synergistic relation with VEGF; it collectively enhances vascular permeability and intensifies the inflammatory response (Zhao et al. 2025). This interaction can initiate a recurrent cycle of endothelial activation, increased leukocyte migration and subsequent vascular damage. Similar patterns were observed in diseases with chronic inflammation and vascular dysregulation, such as sepsis and cancer (Parikh et al. 2006; Park et al. 2007). These findings highlighted not only the potential of Ang‐2 as a biomarker for disease severity but also as a therapeutic target (Gerald et al. 2013). Promising results in reducing vascular leakage and inflammation have been reported with inhibition strategies targeting Ang‐2, either alone or in combination with VEGF blockade, in experimental models (Lopes‐Coelho et al. 2021).

VEGF is a critical regulator of vascular endothelial growth, proliferation and permeability (Ahmad and Nawaz 2022). A significant increase in VEGF levels was observed in the late disease stages. The dogs in G3b probably reflected a defence mechanism triggered by inflammation and hypoxia. This elevation facilitates the recruitment of inflammatory cells to infected tissues by promoting angiogenesis, particularly at active infection sites (Fraga et al. 2012). However, high VEGF levels may disrupt vascular barrier integrity, contribute to tissue oedema and potentially exacerbate disease progression. Elevated VEGF levels in chronic inflammation indicate continuous endothelial cell activation and increased vascular permeability (Paleolog 2009). In addition, the potential role of VEGF inhibitors in angiogenesis regulation and vascular permeability reduction was widely studied in cancer, age‐related macular degeneration and diabetic retinopathy (Ferrara and Adamis 2016). The identification of angiogenic and vascular biomarkers in severe CanL cases highlights the need for targeted therapeutic strategies to address uncontrolled vascular changes.

The findings reported here were aligned with previous research about angiogenesis in dogs (König et al. 2018, 2019). These studies demonstrated the potential of Ang‐2 as a biomarker for endothelial dysfunction and disease severity in dogs with inflammatory and infectious conditions. König et al. (2018) first validated a human Ang‐2 immunoassay for canine plasma samples and endothelial cell cultures in 2018. The validated method was reliable for the detection of Ang‐2 levels in canine samples and also established the relationship between TNF‐α‐induced inflammation and Ang‐2 release from endothelial cells. They reported that Ang‐2 levels in dogs with SIRS and sepsis were significantly higher compared to healthy controls, highlighting Ang‐2's prognostic value as a biomarker (König et al. 2019). Overall, both studies collectively demonstrated that vascular homeostasis could be disrupted by Ang‐2, which antagonizes Ang‐1‐mediated stabilization of the Tie2 receptor, contributing to increased vascular permeability and inflammation.

Acute phase proteins, CRP and ferritin, were significantly elevated, particularly in G3a and G3b. In addition, increased CRP levels, a well‐known biomarker of inflammation, may reflect the acceleration of the inflammatory response parallel to disease progression (Ceron et al. 2018). An elevated CRP level is a common finding in chronic infectious diseases, where systemic inflammation plays a central role in disease pathology (Eckersall and Bell 2010). The significant elevation of serum ferritin levels in dogs with CanL likely confirms its dual role as both an inflammatory marker and a regulator of iron homeostasis in chronic diseases. A high level of ferritin was related to the immune response, eliminating pathogens through macrophage activation and iron sequestration (Pardo‐Marin et al. 2020). These findings aligned with previous literature about systemic inflammation in CanL and highlighted the value of acute phase proteins in monitoring disease progression and severity. The observed elevations in CRP and ferritin levels highlight their potential as diagnostic and prognostic markers in clinical practice. However, CRP cannot be evaluated as a single parameter for the diagnosis of CanL, and it should be in alignment with reliable and cost‐effective diagnostic practices (Malin and Witkowska‐Pilaszewicz 2022).

The clinical findings observed in dogs with CanL were consistent with previous reports (Solano‐Gallego et al. 2009; Paltrinieri et al. 2010; Koutinas and Koutinas 2014; Ceron et al. 2018). The clinical symptoms of CanL reported here include cachexia, lethargy, weight loss and peripheral lymphadenopathy. Additionally, dermatological findings such as hypotrichosis, alopecia, ulcerative dermatitis and onychogryphosis are also common in these dogs. Ocular findings in CanL are keratitis and conjunctivitis. Biochemical analysis of *Leishmania* spp.‐infected dogs revealed significantly elevated urea and creatinine levels in the late stage of the disease, indicating impaired kidney function. Similarly, calcium levels were significantly lower, whereas phosphorus levels were significantly higher in late stages, suggesting metabolic instabilities associated with renal dysfunction. Additionally, the dogs in the late stage of the disease showed significant differences in total protein and albumin levels, with hypoalbuminemia and hyperproteinaemia indicating protein loss due to immune complex deposition and kidney damage (Paltrinieri et al. 2010; Roura et al. 2021; de Sousa et al. 2025). The observed elevation of urea and creatinine levels in dogs with advanced CanL in this study contrasts with the findings of another study (Lopes et al. 2024), which reported a relatively low prevalence of such alterations in a large retrospective cohort. This discrepancy may reflect differences in case selection and disease staging, as well as the known limitations of creatinine and urea as early indicators of renal injury. Although the current data support the association between CanL and renal dysfunction in late stages, such as exemplified in other studies (Pereira et al. 2020), the contrasting findings reinforce the potential role of VEGF, Ang‐1 and Ang‐2 in CanL and the need for more sensitive and specific biomarkers for early renal involvement and CanL diagnosis. These findings support the detrimental impact of CanL on renal function and systemic homeostasis.

The findings of this research provided novel insights for the researchers to examine the role of VEGF, Ang‐1 and Ang‐2 in the pathophysiology of CanL. However, the generalizability of these results is subject to some limitations. First, the relatively small sample size may restrict the generalizability of the findings and limit statistical power for subgroup analyses. Second, the cross‐sectional (single time‐point) design does not allow for assessment of longitudinal or dynamic changes in biomarker levels, precluding conclusions about their temporal evolution or prognostic value. Third, although statistically significant differences in VEGF, Ang‐1 and Ang‐2 were observed among groups, there was partial overlap between individuals, which reduces their discriminatory ability at the individual patient level. Therefore, these markers should not be interpreted as standalone diagnostic or prognostic indicators, but rather as part of a comprehensive clinical and laboratory assessment. Additionally, the long and variable incubation period of CanL and individual differences in inflammatory response make it inherently difficult to sample all animals at the same stage of disease, despite classification efforts based on acute phase proteins and clinical status. Finally, although the use of commercially available ELISA kits provided reliable measurements, intraspecies variability in the sensitivity and specificity of assays remains a potential confounding factor.

## 5 Conclusions

In conclusion, the investigation of serum VEGF, Ang‐1 and Ang‐2 levels in different stages of CanL has shown their potential function in the progression of the disease. Elevated VEGF and Ang‐2 levels possibly revealed increased vascular permeability and inflammation; in contrast, elevated Ang‐1 suggested a compensatory mechanism to maintain vascular stability. These findings may have significant implications for understanding the biomarker potential of these parameters to follow up on the disease severity and develop novel targets for therapeutic applications. However, due to partial overlap among groups and inherent variability in the disease course and host response, these biomarkers should not be interpreted as standalone indicators, but rather as part of a broader clinical and laboratory assessment. Further research in larger cohorts, with more biomarker panels, as well as including imaging techniques for direct evaluation of vascular changes, is needed to generalize and expand the findings of this study.

## Author Contributions

**Gamze Gultekin**: conceptualization, methodology, data curation, writing – original draft, writing – review and editing. **Erdogan Malatyali**: conceptualization, methodology, writing – review and editing. **Hatice Ertabaklar**: methodology, supervision, writing – review and editing. **Evren Tileklioglu**: methodology, writing – review and editing. **Songul Erdogan**: formal analysis, writing – review and editing. **Gamze Sevri Ekren Asici**: data curation, writing – review and editing. **Asude Gulce Oryasin**: investigation, resources. **Serdar Pasa**: supervision, project administration. **Kerem Ural**: supervision, project administration, writing – review and editing. **Hasan Erdogan**: formal analysis. **Mehmet Gultekin**: conceptualization, funding acquisition, writing – review and editing, supervision.

## Funding

This study was partially summarized from the project funded by Aydın Adnan Menderes University Research Projects Funding Unit (Project No.: VTF‐21003).

## Conflicts of Interest

The authors declare no conflicts of interest.

## Data Availability

The authors confirm that the data of this study are available in the article.
